# Does the regional deprivation impact the spatial accessibility to dental care services?

**DOI:** 10.1371/journal.pone.0203640

**Published:** 2018-09-07

**Authors:** Hosung Shin, Eunsuk Ahn

**Affiliations:** 1 Department of Social and Humanity in Dentistry, Wonkwang University School of Dentistry, Iksan, North Jula, Republic of Korea; 2 Department of Dental Hygiene, Kyungbok University, Pocheon, Gyeoggi, Republic of Korea; Western University, CANADA

## Abstract

The aim of the present study was to assess the regional deprivation and individual factors that influence how far a person will travel to access dental care. Using data from the Korea Health Panel (2008 to 2011), we selected a group of 4,256 subjects and geocoded their homes and dental hospitals/clinics. Using the road network analysis, we calculated the distance traveled by the subjects for dental care. We used the generalized estimating equation (GEE) for repeated data analysis and included an interaction term between regional deprivation and individual income to determine the effects of the two factors on the choice of a dental hospital/clinic. When the regional deprivation index was divided into three quarters (high, middle, and low), urban areas had higher”high” and “low" levels of deprivation, and rural areas had relatively higher middle level of deprivation. GEE regression showed that the level of education, regional deprivation level, and income all affected the distance traveled to dental clinics. The regional deprivation level had a higher association than income with the travel distance. At the same income level, subjects who lived in the least deprived areas were more likely to travel longer distances than subjects living in the most deprived areas. Regarding the distribution of dental hospitals/clinics, incentive based dental polices for either dental providers or patients are needed that will assure the delivery of dental care despite spatial inequality.

## Introduction

Accessibility to a dental hospital/clinic, which includes dental clinics, dental hospitals, and dental departments in hospitals, was reported to be closely related with an individual’s socioeconomic status and place of residence, as well as the regional distribution of health care resources [1,2]. Based on the theory of social determinants of health, health care utilization contributes only a portion of its resources to health care outcomes, other factors include life style and health behavior, social economic factors, and social and physical environment [2]. Considering that adequate use of health care services will likely particularly benefit the health outcomes of patients with acute illnesses, improvements in the quality and availability of these services are important goals for health care consumers [3]. The barriers that impede proper health care utilization include economic (i.e., an individual’s ability to pay for services) and access issues (i.e., the availability of health care personnel and facilities) [4,5]. The distribution of health care resources is also an important factor in determining accessibility and availability, and it influences total health care costs and travel times [6].

The health level of a society—such as standardized mortality rate, life expectancy, and disease prevalence etc.—is shaped by not only unavoidable factors that stem from individual characteristics but also by avoidable factors related to individual socioeconomic status or social aspects of the area of residence [3,7]. There is an interaction between the various social conditions and the socioeconomic statuses of the residents [4,5]. The concept that the social and environmental characteristics of a region may be mirrored by its health level has led to studies that have confirmed the so-called “context effect” in health care utilization [8]. In terms of health care utilization, regional characteristics may cause an area to be excluded from the opportunity to receive equal health care benefits [9,10]. Because individual socioeconomic status influences the decisions and choices about health care intervention, individual and regional conditions interact to determine the status of a person’s health.

The characteristics of dental care utilization differ from those of other medical services because oral diseases are rarely life-threatening. Therefore, non-disease-related factors, such as sociodemographic characteristics, have been reported to exert a more pronounced effect on the utilization of dental care [11–14]. Previous studies have demonstrated a difference in dental care utilization based on the area of residence (i.e., urban vs. rural), with lower utilization in rural areas [15–17]. Spatial accessibility, which refers to both physical impedance and use of health care services, has often been measured based on a respondent’s perceived time and distance to healthcare facilities [10,18]. A variety of models were introduced to measure spatial accessibility to healthcare [18,19]. From the perspective of spatial accessibility, Khan [20] has categorized access measures into four dimensions: spatial-potential, spatial-realized, aspatial-potential, and aspatial-realized. Potential dimension measures the availability of healthcare services that allows potential patients to use healthcare services, and realized dimension is related to the actual use of healthcare services by healthcare users. Spatial access refers to geographical access of healthcare, and aspatial access is a social component of an individual or community factors that is not related to the geographical aspect but influences access to medical care. Guagliardo [18] has classified previous spatial accessibility measures into four categories: provider-to-population ratio, distance to the nearest provider, average distance to a set of providers, and gravity-based accessibility.

Spatial accessibility also influences a patient’s perceived willingness and ability to receive health care and actual use of health care services [18,21]. In this study, we sought to identify the regional and individual factors that affect the realized acess of dental care for individuals. We measured the travel distance to dental hospitals/clinics and examined the association of the factors related to the travel distance and individual and regional socioeconomic status on dental care utilization.

## Materials and methods

### Data and subjects

This study used Korea Health Panel (KHP) data from the period 2008 to 2011. The KHP survey provides nationally representative health expenditure data, and it was designed to amass information about health care utilization, health behaviors, medical expenditure amounts, socioeconomic status, and the status of private health insurance for the members of a household [22]. The KHP employed the South Korea Population and Housing Census as its sampling frame and used stratified cluster sampling, with probability proportional to size. The KHP questionnaire items were answered by the head of household on behalf of the entire family. The respondents provided written informed consent to participate in the KHP [22].

Patients tend to frequent health care institutions that are located within their individual sphere of activity. These areas will differ based on individual characteristics and geographic locations. For example, job holders are more likely to select a dental hospital/clinic located near their workplace [3]. Since the data do not provide workplace address of respondents, the study only included students (individuals receiving formal education), and teenagers under 14 years of age, as well as the self-employed, elderly, and economically active population who did not work in offices (i.e., individuals involved in housekeeping, child-rearing, etc.). The final analysis used 5,256 subjects. The study was reviewed and approved by the Institutional Review Board of Wonkwang University, Iksan, South Korea, which also approved this secondary data analysis (WKIRB-201512-SB-048). The research was conducted in full accordance with the World Medical Association Declaration of Helsinki.

### Measurement of travel distance

**Fig 1:**
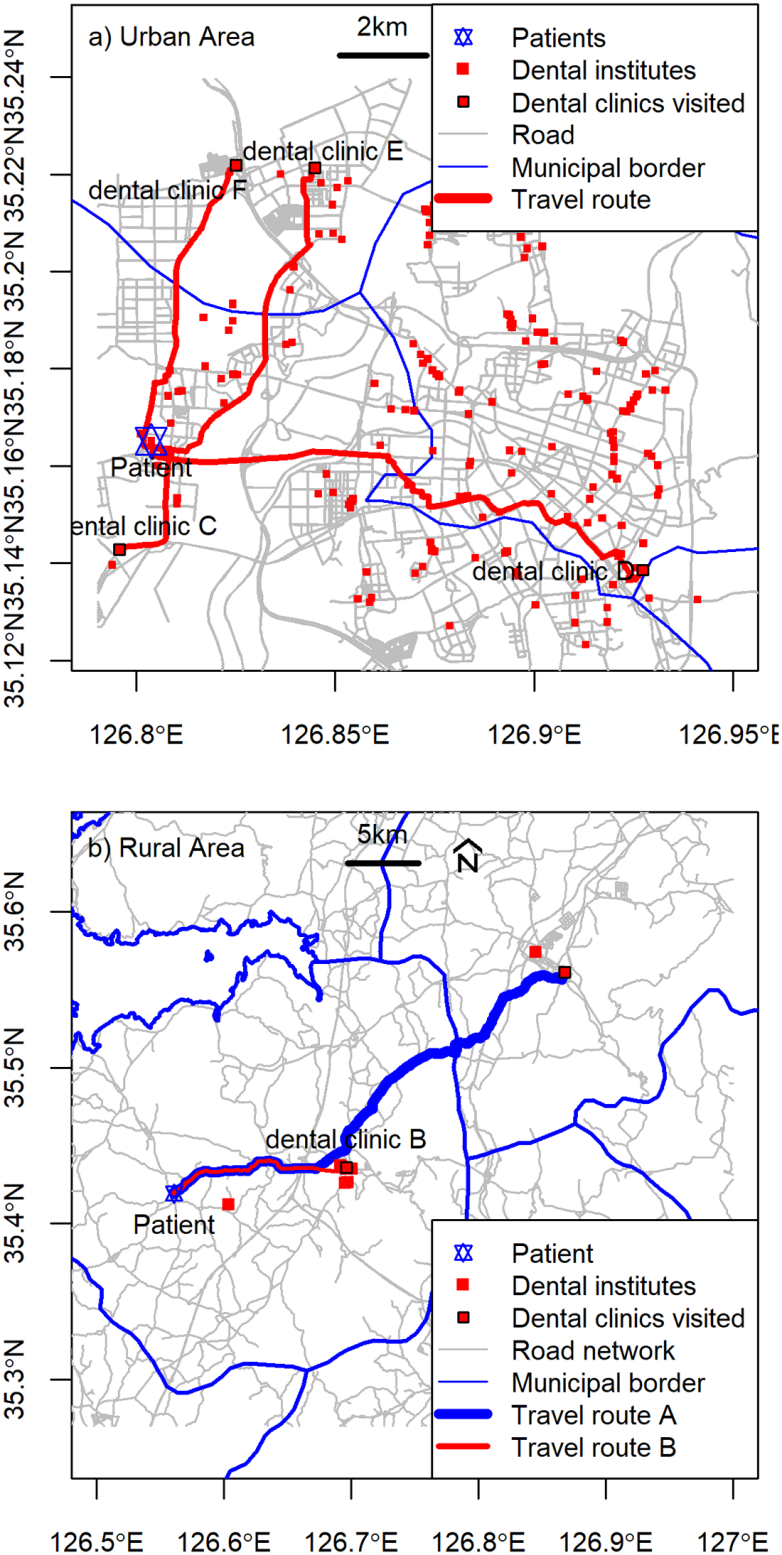
Typical travel distance to dental clinics by region (urban vs. rural). Note: Lines in the figure delineate the travel route to a dental clinic/hospital.

To measure the travel distance, we used road network data, which consisted of annually revised standard node-link data collected by the Korea Transport Institute, a department housed under the Korean Ministry of Land, Infrastructure, and Transport. A “node” expresses the point at which the recommended speed (designated by an authority) of a car changes, and a “link” is the line that connects two nodes, which is a real-world road. The network analysis included three tasks: geocoding, which marked the subject of measurement on a map, network maintenance, which connected the locations of medical institutions and of study subjects to the road network, and identification of the shortest path between two locations. Fig 1 shows the typical travel path and distances from the patients’ residences to the dental clinics. Travel distances in urban areas are usually limited to central areas, whereas those in rural areas extend beyond the boundary of municipal areas. The network analysis was performed using open-source software (GRASS GIS version 6.4).

### Data analysis

Because of the repeated measurements of KHP, we required a model that would consider the intra-class correlation between these repeated measurements. The generalized estimation equation (GEE) described by Liang and Zeger was used for the analysis [23]. Panel data analysis can be divided into a “subject-specific” (SS) model, which explicitly shows heteroscedasticity among subjects, and a “population-averaged” (PA) model, which is represented as a function of covariates without considering the explicit heteroscedasticity among subjects. The PA model focuses on the average of all changes rather than on individual differences among the subjects being analyzed. For the analysis, the study used the PA model. The variables that were considered to be factors related to the selection of dental hospitals/clinics for dental service use were age, education, equivalent income, treatment type, the composite deprivation index (CDI) [24] and region (urban and rural). The travel distance, a dependent variable, had a distribution that was shifted to the right side, and it was included in the analysis following natural log transformation.

$$\begin{array}{l} \text{log}(\text{G}\left(travel\;distance\right))\;=\beta_{1}\text{sex}+\beta_{2}age+\;\beta_{3}education+\;\beta_{4\;}equivelant\;income\\ +\;\beta_{5}type\;of\;treatment+\beta_{6}region+\;\beta_{7}regional\;deprivation\;index\\ +\;\beta_{8}\;\left(equivalent\;income*regional\;deprivation\;index\right) \end{array}$$

Among the individual variables, age was divided into four groups, taking into account the pattern of dental care utilization: (1) 10 years of age and younger, when most treatments are conservative, (2) 11 to 25 years of age, when treatments are chosen by parents who have financial control (although age 20 years old is considered to be an adult, offspring are still under parental economic influence before they graduate from university), (3) 26 to 64 years of age, which includes young adults and middle-aged people, and (4) 65 years of age and older, which includes the elderly. We divided the level of education into two groups: “high school or less than high school graduate” and “college students or college graduate”. The treatment type was divided into two groups, with more expensive treatments, such as dental prosthetics, orthodontics, and implants, in one group, and all other treatments in the other group. Household equivalent income, which represents the total household income and reflects family size, was used as a continuous variable and the unit for equivalent income is a million KWN (900 USD).

South Korea had 17 provinces, 251 municipalities (mean population: about 200,000 [SD = 150,000]), and over 3,500 sub-municipalities (mean population: 13,800 [SD = 12,300]) in 2010–2011. The study classified 251 municipalities into two types, urban (district and middle-to small city) and rural (county), based on the population size and distribution of dental resources. The deprivation index is widely used to indicate the socioeconomic status of a region. The CDI used in this study is composed of five different subdomains (unemployment, poverty [public assistance], housing, labor, and social relations) [24]. The CDI has a theoretical value between 0 and 500; a higher score correlates with a more deprived region. Initially, we divided the regional CDI into five quintile groups, with the high level being 5, the low level being 1, and the middle level being 2, 3, and 4; however, we later counted the middle level as a single, combined group, resulting in a total of three groups. The reason for classifying three groups was to observe the impact of income and CDI has on dental health care utilization rather than dose-response effect.

In the final analytical model, we included an interaction term (equivalent income * regional deprivation) to examine the total effect of income and CDI on the distance traveled to a dental hospital/clinic and to assess how the impact of income on travel distance differs from the level of CDI or vice versa. To determine the interaction effect, it was necessary to control for the possibility of multicollinearity between interaction terms and the variables used for the interaction. In addition, to clarify the interpretation of regression coefficients, mean-centered interaction variables ($X-\overset{-}{X})$ were included. ANOVA (including Bonferroni test), t-test, chi-square test, and Mood’s median test was used for descriptive analysis.

## Results

The analysis of 4,256 subjects who received dental care at least once in the previous 4 years revealed an average number of six treatments per subject. The general characteristics of dental care users from 2008 to 2011 are shown in Table 1, according to region. Most treatments were conservative (76%), and in the case of prosthetics, the proportion was slightly higher in urban areas (25%) than in rural areas (23%). The urban areas had high distributions of both high and low CDI scores, whereas the middle scores were high at 62% in rural regions. In a comparison of individual income levels, there was a higher proportion of high-income earners in urban regions compared with rural regions.

**Table 1:** Distribution of general characteristics (by region) based on dental care utilization (2008–2011).

		Urban (n = 24,199)	Rural (n = 8,199)	Total (n = 32,398)
**Sex**	**Male**	34.61	37.16	35.26
	**Female**	65.39	62.84	64.74
**Age group**	**Younger than 10**	19.40	20.76	19.74
	**10 to 24**	31.82	27.55	30.77
	**25 to 64**	31.49	28.64	30.74
	**65 years and older**	17.29	23.05	18.75
**Treatment type**	**Conservative**	77.09	74.53	76.44
	**Prosthetic and others**	22.91	25.47	23.56
**CDI**	**High**	21.96	9.84	19.94
	**Middle**	56.33	78.64	60.04
	**Low**	21.71	11.52	20.01
**Equivalent income (mean±SD)**^a^		2.13±1.43	1.80±1.25	2.10±1.42
**Level of educational attainment**	**Others**	81.20	87.48	81.69
	**College**	18.80	12.52	18.31
**Average number of dental visits (mean±SD)** ^b^		6.08±6.85	5.99±7.90	6.05±7.13
**Travel distance to dental hospital/clinic**	**Mean±SD (km)**	7.74±29.86	14.01±23.80	8.23±29.48
	**Median (km)**	1.65	7.27	1.75

CDI: composite deprivation index.

^**a**^ The unit of equivalent income is 10 million KWN (9,090 USD)

^**b**^ All variables except the average number of dental use per respondent were statistically significant at p-value 0.05

**Fig 2:**
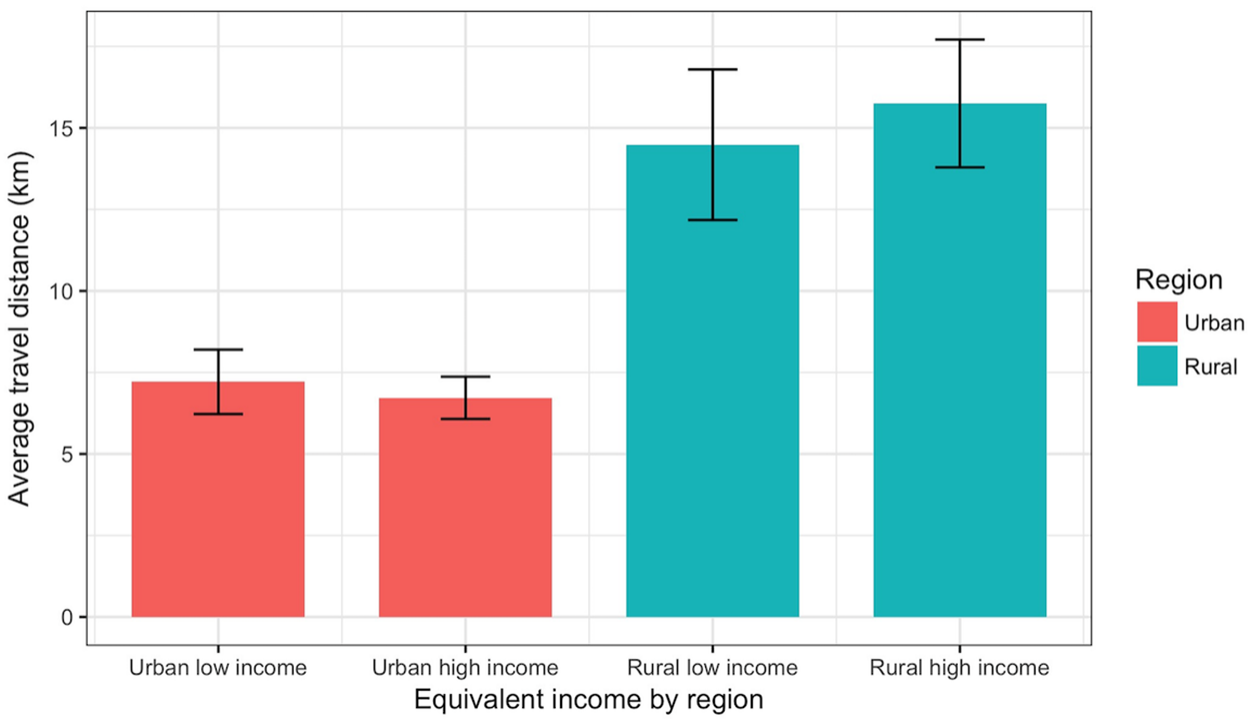
Travel distance to dental institutions, according to urbanization and the household equivalent income.

Fig 2 shows the travel distance when selecting a dental hospital/clinic, according to the type of municipality and the household equivalent income. According to the level of equivalent income, the distance from households to visited dental clinics was different in urban and rural areas. In rural areas, respondents with the high equivalent income group tend to travel long distances compared to respondents with the low equivalent group (p < 0.001), while urban respondents visit shorter distance dental clinics for dental services (P < 0.001), respectively.

Table 2 shows the regional and individual factors that associate travel distance when selecting a dental hospital/clinic, according to three models. In Model 1, which only considered regional factors, the travel distance was not significantly associated by the degree of regional deprivation, but the subjects traveled 2.56 times farther in rural regions than in urban regions. In Model 2, which considered individual factors alone, the age groups spanning 10 to 24 years of age and 65 years of age and older traveled 1.2 times farther compared with the reference group (25 to 64 years old). The results for the age group 65 years of age and older can probably be attributed to the characteristics of the elderly, such as a generally poor financial status, retired and having an increased need for rehabilitative care such as prosthetic treatment.

**Table 2:** Regression analysis results.

Model 1			Model 2			Model 3		
	IRR	95% CI		IRR	95% CI		IRR	95% CI
**CDI**			**Sex**			**Sex**		
**Middle**	0.977	(0.872–1.093)	**Female**	1.048	(0.960–1.145)	**Female**	1.025	(0.936–1.123)
**Low**	0.893	(0.780–1.204)	**Age**			**Age**		
**Region**			**Younger than10**	0.945	(0.903–0.990)	**Younger than10**	0.937	(0.920–0.954)
**Rural**	2.559	(2.278–2.284)	**10–24**	1.192	(1.096–1.298)	**10–24**	1.303	(1.233–1.377)
			**65 years and older**	1.199	(1.082–1.328)	**65 years and older**	1.029	(0.961–1.102)
			**Type of treatment**			**Type of treatment**		
			**Prosthetics others**	1.195	(1.172–1.219)	**Prosthetics others**	1.167	(1.159–1.176)
			Equivalent income*	1.040	(1.029–1.051)	Equivalent income^*^	1.027	(1.015–1.040)
			**Education**			**Education**		
			**College**	1.115	(1.042–1.193)	**College**	1.095	(1.064–1.128)
			**Region**			**Region**		
			**Rural**	2.173	(1.970–2.387)	**Rural**	2.665	(2.373–2.993)
						**CDI**		
						**Middle**	0.973	(0.869–1.089)
						**Low**	0.857	(0.748–0.982)
						**CDI×Income**		
						**Middle**	1.000	(1.000–1.000)
						**Low**	1.003	(1.000–1.000)

IRR = incidence rate ratio; CI = confidence interval; CDI = composite deprivation index; CDI = Income denotes the interaction term between CDI and income.

Asterisk (*) indicates that the unit for equivalent income is 10 million KWN.

According to the treatment type, the group requiring prosthetic and other expensive treatments (e.g., orthodontia and implants, among others) traveled 1.2 times farther than the group requiring conservative treatment (e.g., periodontal treatment and preventive care). The regression results showed a 3% increase in the distance traveled per 10 million KWN, meaning that income had little impact on the distance traveled, given that the average equivalent annual income of the respondents was 21 million KWN. In addition, the “college student or college graduate” group showed a 1.12-fold increase in the distance traveled compared with the “high school or less than high school graduate” group.

Model 3 considered both regional and individual factors. The effects of the individual characteristics of the respondents did not differ greatly from Model 2, but the effect of regional deprivation changed significantly. The CDI had a higher association than individual income on the distance traveled. The interaction between individual income and the CDI was statistically significant, indicating that the effect of income differs based on the degree of regional deprivation. The travel distance tended to increase in alignment with the increase in personal equivalent income. This serves to increase effects of equivalent income in the least deprived area compared to the most deprived areas (reference group). Considering these interactions, the total effect of the equivalent income showed that the travel distance increased by 3.2% in the least deprived areas, while that of the most deprived was 2.7% every 10 million KWN increase.

## Discussion

In this study, we examined the factors that associate with the travel distance (at the regional and individual levels) of an individual choosing a dental hospital/clinic. It was observed that several individual factors, as well as the degree of regional deprivation, was associated with travel distance. These factors included the treatment type, education level, and regional deprivation. When an individual utilizes dental care services, the cost includes the price of the service as well as the opportunity cost of the traveled distance. Thus, travel distance can be considered as the geographic proximity between the patient’s residence and the dental hospital/clinic, and also as a factor for the selection of a specific dental hospital/clinic based on the regional distribution of the dental hospitals/clinics and personal preference.

In Korea, National Health Insurance (NHI) dental coverage was raised by a small amount recently, and dental implants and denture services for the elderly were included in the NHI dental plan. However, in 2008–2011, which this study focuses on, the dental care system charged relatively higher out-of-pocket payments (about 70% in the NHI report, and more than 80% in the 2011 OECD Health Data) compared with other general medical services (about 30% out of pocket), and most expensive services were not covered by government insurance [25]. For this reason, accessibility varies based on individual socioeconomic status, and the geographical distribution of dental institutions also influenced physical accessibility.

Compared with other treatment types, the travel distance tended to increase for relatively more expensive treatments, such as dental implants, prosthetics, and orthodontic treatment. Such treatment type differences might be attributable to an effort and wish to receive better dental services for value for money, which increased the willingness of patients to travel greater distances to dental clinics. These differences also indicated that although near and distant dental clinics were included in a resident’s potential travel range, spatial obstacles were much reduced for these expensive dental services. These results are similar to those reported by Shiikha et al. [17], who stated that “Patients with special needs may find access to dental care problematic, especially if they do not reside near a health care organization”.

When comparing residence areas, rural regions had a longer travel distance compared to urban regions, likely because there are fewer dental hospitals/clinics in rural regions. This result is consistent with those of previous studies [7,12]. In rural areas, the distance and duration of trips to health care facilities can be obstacles to follow-up visits [26]. Moreover, compared to urban dwellers, rural residents tend to be burdened by more opportunity costs in utilizing dental care services [26,27]. This study showed that rural residents tend to travel approximately 2.7 times father than city residents.

Previous studies examining personal income or education level reported that a proportional relationship is typically observed in the level of dental care utilization [28]. However, after deciding to receive treatment, the travel distance of a dental hospital/clinic is influenced by other factors. When considering the degree of regional deprivation alone, as in *Model 1*, this variable had no significant effect on travel distance. This null result was due to the dominant impact of urbanization, and small differences within the same regional type were not significant. However, after including individual factors and interaction terms, as in *Model 3*, the degree of regional deprivation demonstrated a significant association with the travel distance. Household equivalent income level alone affected travel distance, but the absolute level of association was negligible (an increase of 10 million KWN led to just a 2.7% increase in the travel distance), whereas the travel distance of a dental hospital/clinic was significantly altered by regional characteristics and other social factors. The average yearly income of the high-income group living in highly deprived regions, for example, was 37 million KWN, and the average yearly income of the low-income group living in less deprived regions was 3.4 million KWN. Patients living in regions with a high degree of deprivation tended to visit dental hospitals/clinics far outside their normal area of activity. The travel distance for low-income earners living in less deprived regions was even shorter than that of high-income earners living in highly deprived regions. The median travel distances for both groups were 1.3 km and 1.5 km, respectively. However, the difference between the two groups might exceed the average 0.2 km when considering the perspective that the distance includes the opportunity cost of traveling a longer distance.

From the perspective of travel distance, the selection of a healthcare institution was related to the establishment of a healthcare service delivery system within the area of individual activity. Martin [29] has defined the area of activity as all places in which a group of people reside and pursue their life goals. Areas of activity include the shopping sphere, commuting sphere, and medical service sphere, where a person can receive medical treatment. Kilaru et al. [30] have stated that the healthcare service area can be defined as an area in which health care service is influential and can be differentiated from other general business districts. Healthcare service areas can be divided into the catchment area, an area controlled by the central or local government, and the service area, where market power is exercised. In Korea, the location of dental hospitals/clinics is highly correlated with the area of activity. Thus, the supply of dental services in large cities is often excessive, whereas supply is below average in rural areas. Dental resources are unevenly distributed, leading to inefficient utilization and unequal accessibility. To establish an appropriate service delivery system to provide dental care, this problem needs to be viewed from the perspective of the access to dental care within a person’s sphere of activity.

The present study measured the actual travel distance of patients who visited dental hospitals/clinics and observed the influence of individual and regional factors on this measured distance. A limitation of this study was the exclusion of workers; KHP did not provide any information on their workplaces, therefore, we could not geocode the workplaces or calculate the travel distance from the workplace to a dental hospital/clinic. The travel distance to a dental hospital/clinic was influenced by regional factors, and even after restricting the geographical factors (e.g., degree of urbanization), the travel distance tended to increase according to the degree of deprivation. The results of this study suggest that the distribution of dental resources should be similar to an individual’s daily sphere of activity to overcome spatial barrier and to provide patient oriented dental care. Taking these factors into consideration, our results may help to improve the unequal distribution of dental care resources.

## Supporting information

S1 File plos_distance.dta. (DTA)
